# Future requirements for and supply of ophthalmologists for an aging population in Singapore

**DOI:** 10.1186/s12960-015-0085-4

**Published:** 2015-11-17

**Authors:** John P. Ansah, Dirk De Korne, Steffen Bayer, Chong Pan, Thiyagarajan Jayabaskar, David B. Matchar, Nicola Lew, Andrew Phua, Victoria Koh, Ecosse Lamoureux, Desmond Quek

**Affiliations:** Signature Program in Health Services and Systems Research, Duke-NUS Graduate Medical School, 8 College Road, Singapore, 169857 Singapore; Singapore National Eye Centre, 11 Third Hospital Ave, Singapore, 168751 Singapore; Institute of Health Policy & Management, Erasmus University Rotterdam, Burg Oudlaan 50, 3000 DR Rotterdam, Netherlands; Department of Medicine, Duke University Medical Center, Durham, NC 27710 USA; Duke-NUS Graduate Medical School, 8 College Road, Singapore, 169857 Singapore; Singapore Eye Research Institute, 20 College Road, Singapore, 169856 Singapore

**Keywords:** Workforce projections, System dynamics, Simulation modeling, Ophthalmologists, Singapore

## Abstract

**Background:**

Singapore’s population, as that of many other countries, is aging; this is likely to lead to an increase in eye diseases and the demand for eye care. Since ophthalmologist training is long and expensive, early planning is essential. This paper forecasts workforce and training requirements for Singapore up to the year 2040 under several plausible future scenarios.

**Methods:**

The Singapore Eye Care Workforce Model was created as a continuous time compartment model with explicit workforce stocks using system dynamics. The model has three modules: prevalence of eye disease, demand, and workforce requirements. The model is used to simulate the prevalence of eye diseases, patient visits, and workforce requirements for the public sector under different scenarios in order to determine training requirements.

**Results:**

Four scenarios were constructed. Under the baseline business-as-usual scenario, the required number of ophthalmologists is projected to increase by 117% from 2015 to 2040.

Under the current policy scenario (assuming an increase of service uptake due to increased awareness, availability, and accessibility of eye care services), the increase will be 175%, while under the new model of care scenario (considering the additional effect of providing some services by non-ophthalmologists) the increase will only be 150%. The moderated workload scenario (assuming in addition a reduction of the clinical workload) projects an increase in the required number of ophthalmologists of 192% by 2040.

Considering the uncertainties in the projected demand for eye care services, under the business-as-usual scenario, a residency intake of 8–22 residents per year is required, 17–21 under the current policy scenario, 14–18 under the new model of care scenario, and, under the moderated workload scenario, an intake of 18–23 residents per year is required.

**Conclusions:**

The results show that under all scenarios considered, Singapore’s aging and growing population will result in an almost doubling of the number of Singaporeans with eye conditions, a significant increase in public sector eye care demand and, consequently, a greater requirement for ophthalmologists.

**Electronic supplementary material:**

The online version of this article (doi:10.1186/s12960-015-0085-4) contains supplementary material, which is available to authorized users.

## Background

The purpose of this paper is to project, up to 2040, the requirements for and supply of ophthalmologists for Singapore under plausible future scenarios. In accomplishing that, we aim to produce credible estimates of the prevalence of eye diseases and the demand of eye care service, as well as how the demand for services translates into workforce requirements and supply.

Several factors highlight the importance of a timely assessment of future needs for the eye care health workforce in Singapore. Singapore is undergoing a significant demographic change, which will affect future eye care service needs. The resident population of Singapore is aging, and the prevalence of several chronic eye conditions has been shown to increase with age [1]. In Singapore, the population aged 65 and above is projected to rise by 207% from 2010 to 2050 [2]. This demographic shift, combined with population growth and increasing life expectancy, is likely to lead to a substantial increase in eye disease and demand for eye care. The demand for services is also influenced by factors such as technological innovations (pharmaceuticals, diagnosis and treatment equipment, and techniques) and organizational innovations that aim to improve the performance of healthcare systems in addition to the potential changes in eye care service demands due to a more highly educated elderly population having greater expectations and awareness of services. The aging Singaporean population also presents patterns of needs that require a shift towards services to manage chronic eye conditions. This entails adjustment in the composition of the eye health workforce and in their required skill sets.

Demand and workforce projections are also a crucial first step to design policies to address the “unmet need” for eye care in Singapore [3]. Given the training delay for healthcare workforce professionals such as ophthalmologists (who spend 5 years in undergraduate medical school to first become house officers then another 5 years to specialize in ophthalmology), early planning for workforce training needs is essential.

### Eye care services in Singapore

In Singapore, a city-state in Southeast Asia with a population of 5.4 million [4], people with eye care needs can seek specialist eye care services either at public sector specialist outpatient clinics (SOCs) or at private eye clinics located across the country. All ophthalmologists are trained in the public sector. A very small number of ophthalmologists (six in 2012) transition from the public to the private practice sector.

**Figure 1:**
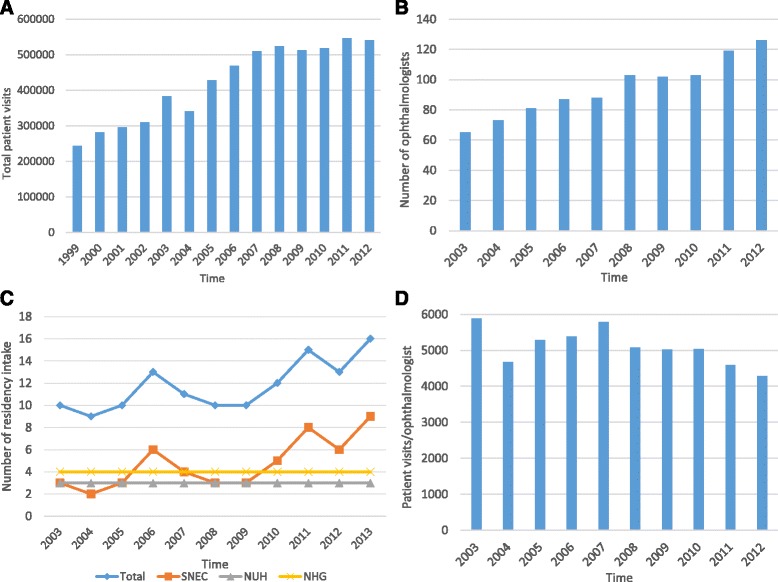
**Singapore public sector eye care workforce background information.** **a** Patient visits, **b** number of ophthalmologists, **c** ophthalmology residency intake, and **d** workload of ophthalmologists (patient visits).

This study focuses on the provision of eye care services in the public sector. Currently, six public hospitals and one specialist eye center (Alexandra Hospital, KK Hospital, Changi General Hospital, National University Hospital, Tan Tock Seng Hospital, Khoo Teck Puat Hospital, and the Singapore National Eye Centre) provide specialist eye care services. Within the public sector, patients may be subsidized or non-subsidized. The Singapore National Eye Centre (SNEC), which began operations in 1990 as a one-stop eye service hub, provides care for about 48% of all public sector eye care visits in Singapore [5]. All seven public eye care centers provide both basic and specialized eye care services and treat a wide variety of eye problems. From 1999 to 2012, the number of patient visits for eye services in the public sector more than doubled from about 240 000 in 1999 to about 540 000 in 2012 (Figure 1a). In parallel, eye care workforce numbers have been rising over the years (Figure 1b)^a^. In this paper, we focus on ophthalmologists although our study also included projections of the workforce requirements for medical officers, optometrists, nurses, technicians, and ophthalmic assistants.

The SNEC, National University Hospital (NUH), and the National Healthcare Group (NHG), which runs Tan Tock Seng Hospital, train ophthalmologists. The number of ophthalmology trainees taken by NUH and NHG has remained almost constant at three and four each from 2003 to 2013; however, intake by SNEC has varied from two to nine over the same time frame (Figure 1c).

Workload, measured by taking total patient visits (utilization) and dividing this number by the numbers of staff in each profession, has decreased over time (Figure 1d).^b^ This may be due to staff seeing more complicated cases or providing a higher quality of care that requires more time, as well as expanding their job scopes to cover other tasks, which may include research, teaching, and administration. These would result in staff seeing fewer patients in a similar period of time. Ophthalmologists in the public sector spend on average 85% of their work hours doing clinical work, 9% doing research, 3% on teaching, and 3% on administration.

## Methods

The Singapore Eye Care Workforce Model was created as a continuous time compartment model with explicit workforce stocks using the systems modeling methodology of system dynamics [6–8]. The model consists of interacting sets of differential and algebraic equations developed from a broad range of relevant empirical data. The systems modeling methodology is well suited to address the dynamic complexity that characterizes health workforce planning [9, 10]. Senese et al. [11] developed a system dynamics model to projects the evolution of the supply of medical specialists. Ishikawa et al. [9] used system dynamics methodology and model to forecast future needs for clinical physicians and OB/GYN specialist and estimated the likely shortfall. Barber et al. [12] created a demand/need simulation model for 43 medical specialists using system dynamics.

The Singapore Eye Care Model was developed to evaluate at the national level demand for eye care services and the implication of service demand for the future workforce and training requirements in the public sector. First, a conceptual computer model that simulated the current behavior pattern of key variables was developed. Next, the conceptual model was presented to the modeling team, which consisted of ophthalmologists, senior nurses, healthcare planners and managers, and health educators from SNEC, to verify its structure and assumptions regarding causal relationships. Following verification, the model was parameterized using data. When data were not available, estimates from experts were used. Finally, the model was simulated, and base-case and alternative scenario projections were made. The insights gained were presented and shared with the modeling team and the strategic planning committee of SNEC including its medical director and chief operating officer, senior ophthalmologists, senior nurses, and researchers.

Generally, health workforce projections employ supply-based methods [13–15] or demand-based methods [15–18]. The sources cited provide more information on the different approaches [19, 20].

Compared with the supply-based and demand-based forecasting approaches, our comprehensive/integrated approach combines the supply-based and demand-based methods to project the eye health workforce needs.

### Singapore Eye Care Workforce Model

The model was constructed as three linked modules: the prevalence of eye disease module, the demand module, and the ophthalmologist requirement and supply module.

#### Prevalence of eye disease module

**Figure 2:**
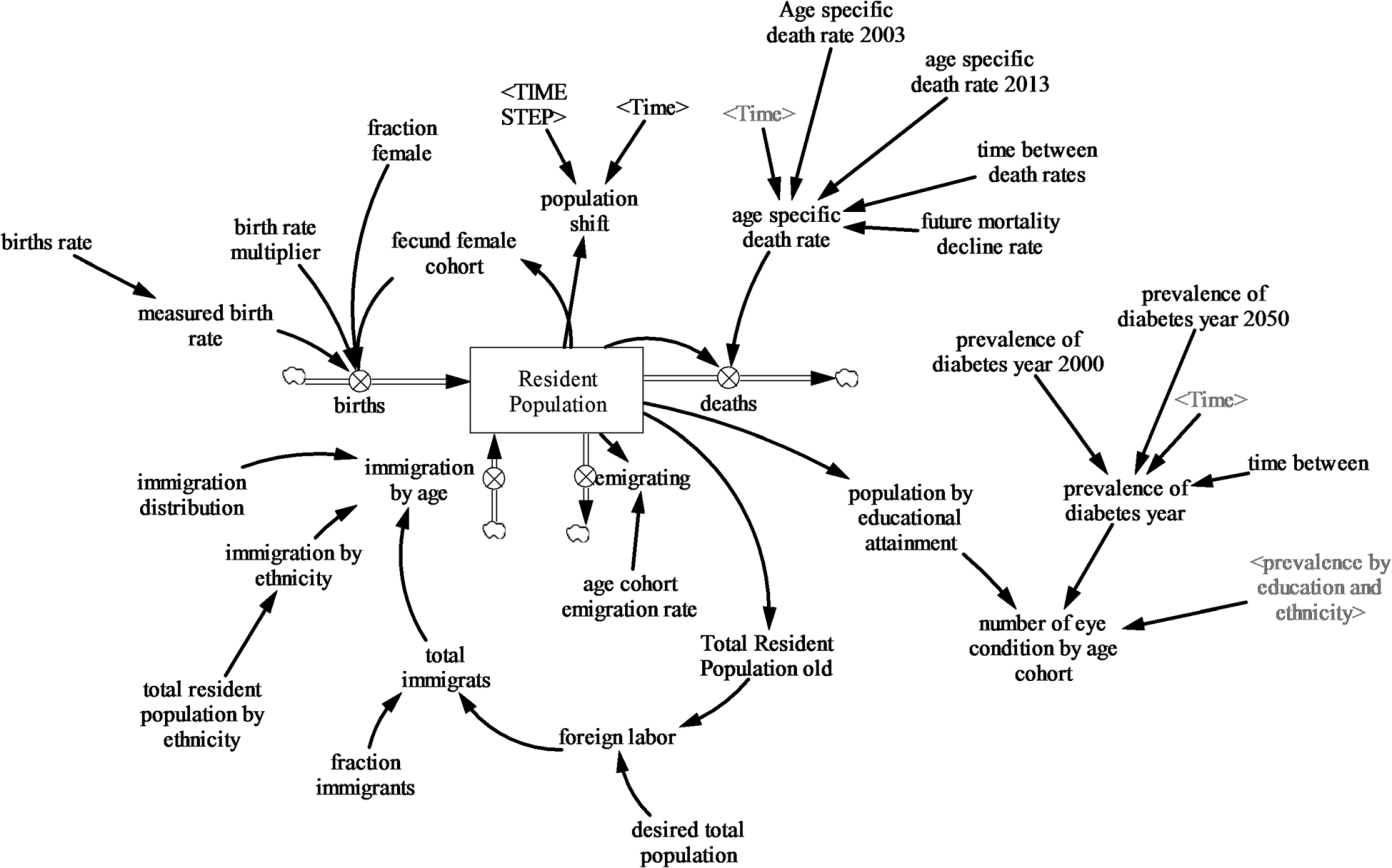
**Prevalence of eye disease module.**

The prevalence of eye disease module applies the prevalence of eye diseases from the Singapore Epidemiology of Eye Diseases (SEED) study [21–23] for resident Singaporeans 40 years and older to the population model of resident Singaporeans (Figure 2). The prevalence of eye diseases was disaggregated by age (age 0–age 100 and older), ethnicity (Chinese, Malays, Indians, and others), and educational attainment (no formal education, primary school, secondary, and tertiary). The eye conditions included herein are cataracts, diabetic retinopathy (DR), glaucoma, age-related macular degeneration (AMD), myopia, refractive error^c^, epiretinal membrane (ERM), retinal vein occlusion (RVO), and other conditions.^d^ The resident population module illustrates an aging process of Singapore’s resident population and the age distribution of the resident population disaggregated by 1-year age cohorts by gender, ethnicity, and educational attainment. The resident population module shows births, deaths, immigration, and emigration as the four determinants of population change over time. It is calibrated using publicly available national statistical data [24] and is described in detail elsewhere [25–27].

To project the number of Singaporeans and permanent residents over 40 with eye conditions, the prevalence estimates from the SEED study was applied to the projected population of residents over 40 years of age till 2040. The projection is disaggregated by age, ethnicity, educational attainment, and eye conditions. The projection for DR takes the expected future increase in diabetes into account [28]. Due to a lack in data, it was not possible to project the prevalence of eye diseases among the younger population; however, with the exception of myopia, the prevalence of eye disease among the young is likely to be much lower than that among the population over 40 years.

#### Demand module

**Figure 3:**
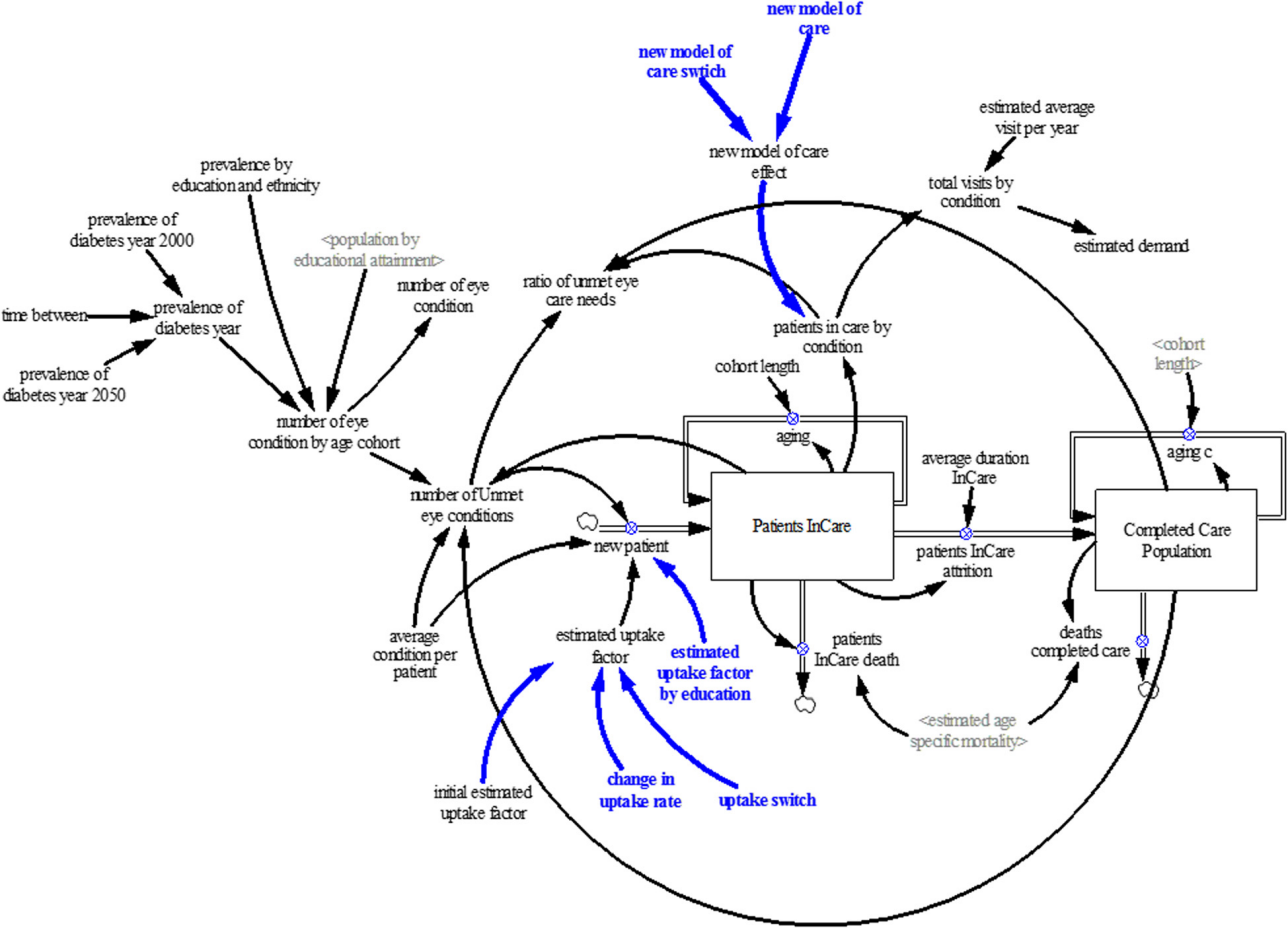
**Demand module.**

The demand module (Figure 3) uses output from the prevalence of eye disease module to project demand for eye services. For this module, we are interested in the demand for public sector specialist eye care services. While the majority of patients at public sector eye care centers are Singaporean and permanent residents over the age of 40, younger people and foreigners also use these centers. Moreover, Singaporeans and permanent residents over 40 will also seek care in the private sector.

We have taken the current number of demand (visits and waiting list) in the public sector (disaggregated by disease according to administrative case mix data from SNEC) as a starting point. Based on the assumption that the change in number of all new patients seeking care will be proportional to the change in the prevalence of conditions among residents, we can project the number of new patients entering the system over the time frame of the simulation. Although foreigners and younger people represent only a comparatively small part of all patients at public sector eye centers, there is a degree of uncertainty connected with this assumption; hence, a sensitivity analysis was performed.

We define the relationship between the number of new patients entering care and the number of Singaporean and permanent residents of age 40+ with untreated eye diseases as (disease-specific) uptake factor. This uptake factor therefore accounts for the visits by foreigners and young people as well. It was estimated by calibration. The uptake factor multiplied by the 40+-year-old resident population in Singapore with eye conditions not receiving eye care services therefore estimates the number of new patients seeking care.

The stock of patients in care increases via new patients seeking care and decreases via death and patient in care attrition. Thus, new patients flow into the age cohorts of patients in care, while the stock of surviving patients in care flows into the subsequent age cohort with the exception of the final age cohort (age 100 and older). Those who have completed treatment flow out to the completed care population stock. The non-surviving patients in each age cohort are removed via deaths—reflecting age-specific mortality. New patients represent individuals seeking care for the first time, whereas attrition of patients in care represents those who have completed treatment in the specialist centers (potentially to seek further care in the community). The completion of treatment is applicable only to cataracts, myopia, and refractive error, with estimated treatment duration at the public specialist eye care centers of 3, 1, and 2 years, respectively. All other eye conditions are assumed to require lifelong care in the specialist eye centers. Mortality rates of patients in care were determined by age-specific mortality from life tables. Attrition of patients in care is calculated as patients in care divided by average duration in care. The completed care population increases via attrition of patients in care and decreases via death. Attrition of patients flow into the population of completed eye care treatment, while the surviving individuals flow into the subsequent cohort with the exception of the final age cohort—age 100 and older. The non-surviving individuals in each age cohort are removed via deaths, reflecting the age-specific mortality.

The demand was calculated from the number of patients in care and average visits per year. The completed care population stock consisted of individuals who have completed eye care treatment for cataracts, myopia, and refractive error.

#### Ophthalmologist requirement and supply module

**Figure 4:**
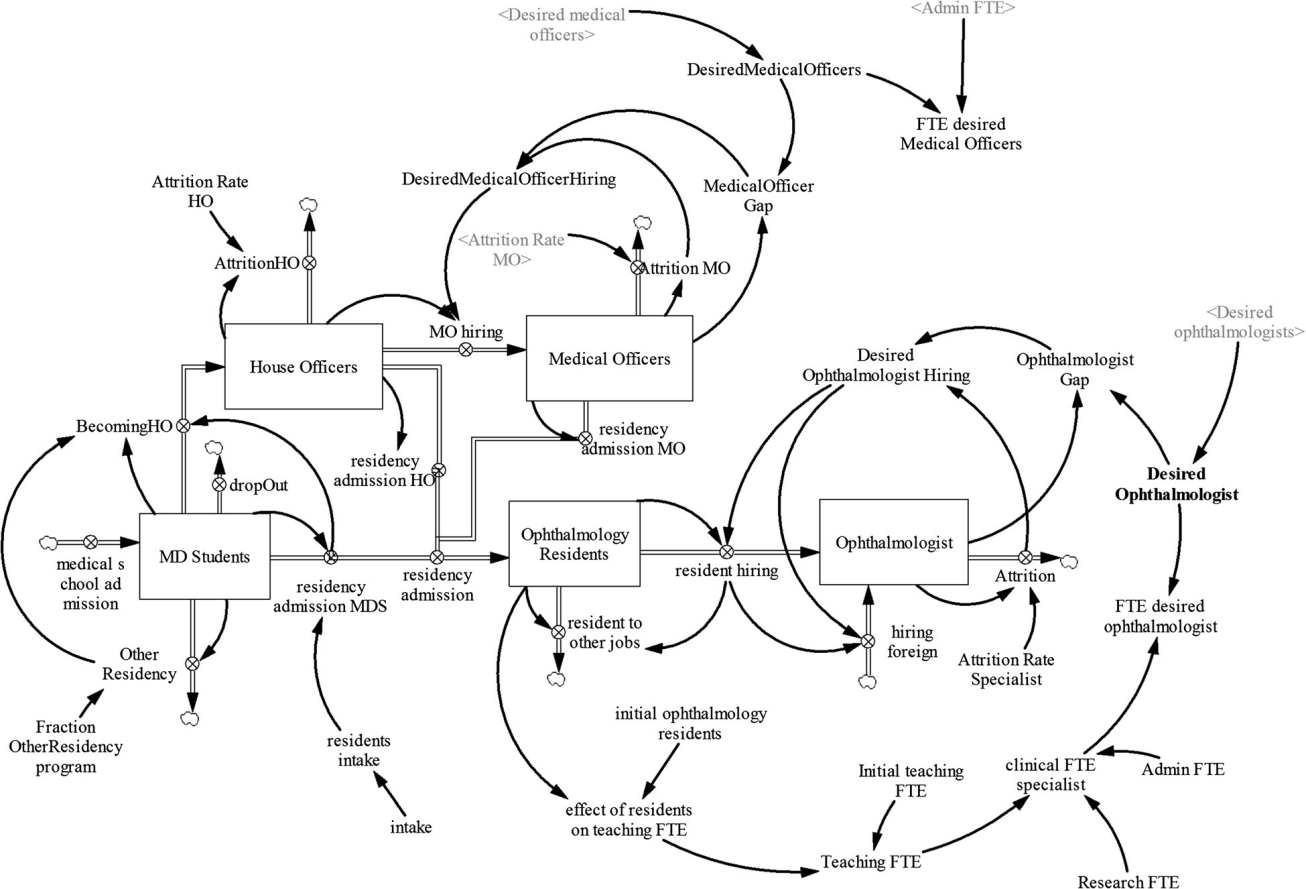
**Ophthalmologist requirement and supply module.**

The ophthalmologist requirement and supply module (Figure 4) is a continuous time compartment model that tracks the demand and the changing number of ophthalmologists employed over time in the public sector, as well as the training pipeline of ophthalmologists. The change in the number of ophthalmologists is a result of new hires and attrition, which is a blended value of retirements, deaths, and resignations. The ophthalmologist requirement module produces three main outputs: the required ophthalmologists, training pipeline of ophthalmologists, and ophthalmologists working in the public sector. The model accounts for sources of recruitments to ophthalmology residency, the training pipeline, decisions for hiring, and the demand-supply gap of ophthalmologists.

As represented in the module diagram, the stock of medical students increases via admissions and decreases via dropout and graduation. Graduating medical students flow out of the medical student stock through three flows: becoming house officers, entering ophthalmology residency, and entering other residency. The source of students entering medical school was considered outside the model boundary. In other words, the source of students suitable for starting medical school was not constrained, and so, it was not necessary to represent schooling prior to medical school.

Thus, the stock of house officers increases as new medical school graduates become house officers and decreases via attrition (becoming general practitioners, entering other residency programs, or taking other jobs), entering the ophthalmology residency program, or becoming medical officers after years of service as house officers.

Likewise, house officers becoming medical officers will eventually decide to remain as medical officers, leave the system for other opportunities, or enter the ophthalmology residency program. Thus, the stock of medical officers increases as new medical officers are hired and decreases via attrition and admission to ophthalmology residency. The transition from house officer to medical officer is determined by the difference between demand for medical officers and current medical officers. Accordingly, medical officer hiring is dependent on desired medical officer hiring. Desired medical officer hiring is defined herein as the medical officers’ gap (difference between desired medical officers and medical officers employed) and expected attrition of medical officers.

The stock of ophthalmology residents (representing the training pipeline) increases as new residents are admitted and decreases via completing the residency program. Upon completion, residents exit the residency stock via hiring to become ophthalmologists or take up other jobs (i.e., become ophthalmologists in the private sector, migrate or take other jobs). The ophthalmology residency intake is represented in the module as a policy variable determined yearly by policy makers. The stock of ophthalmologists integrates new hires (ophthalmology graduate hiring) and attrition (deaths, retirement, quit for other opportunities). Ophthalmologist hiring is determined by desired ophthalmologist hiring, which is defined as the sum of ophthalmologist gap and the expected attrition of ophthalmologist.

On the demand side, the model uses demand for eye care services to project required ophthalmologists. The future ophthalmologist requirement is determined herein by total demand and the estimated workload per ophthalmologist. The workload per ophthalmologist (patient-visits-to-ophthalmologist ratio) is calculated using data from 2003 to 2014.

### Data sources

Demographic data used as inputs for the population module were obtained from the Singapore Department of Statistics (SDS) [24]. Time series data of the resident Singapore population from the SDS were used to calibrate the simulation result of the population module. Age-specific prevalence estimates from the SEED study [21–23] were used. The Ministry of Health (MOH) provided administrative patient visit data disaggregated by patient type (i.e., subsidized and non-subsidized) for the six public hospitals and SNEC in Singapore. SNEC provided administrative patient visit case mix data, disaggregated by age, eye disease, and ethnicity, as well as data from 2003 to 2013 on the numbers of each type of healthcare worker employed by SNEC, and data on ophthalmologists’ work schedule used to estimate the proportion of time spent on clinical work, research, teaching, and administration duties. Data on the number of ophthalmologists in Singapore were obtained from Singapore Medical Council annual reports from 2003 to 2014 [29]. Registration records of ophthalmologists were used to identify their current workplaces. The various data sources and model input parameters are listed in Table 1.

**Table 1:** **Model input**

**Variable name**	**Value**	**Unit**	**Source**
Resident population module			
Birth rate	Time series [2000–2013] by ethnicity	Dimensionless/year	Singapore Department of Statistics
Birth rate multiplier	1	Dimensionless/year	Expert opinion
Age-specific death rate	Time series [2003–2013]	Dimensionless/year	Singapore Department of Statistics
Fraction immigrating	0.3	Dimensionless	Model calibration
Fraction female	0.5	Dimensionless	Singapore Department of Statistics
Fraction male	0.5	Dimensionless	Singapore Department of Statistics
Desired total population	6.9 million	People	Population white paper
Demand module			
Uptake factor [no education]	0.045	Dimensionless/year	Model calibration
Uptake factor [primary education]	0.07	Dimensionless/year	Model calibration
Uptake factor [secondary education]	0.076	Dimensionless/year	Model calibration
Uptake factor [tertiary education]	0.15	Dimensionless/year	Model calibration
Average duration in care			
Cataracts	3	Year	Expert opinion
Myopia	1	Year	Expert opinion
Refractive error	2	Year	Expert opinion
Age-specific mortality rate	Time series [2003–2013]	Dimensionless/year	Singapore Department of Statistics
Distribution of patients in care			
Cataracts	0.31	Dimensionless	Case mix study at SNEC
DR	0.09	Dimensionless	Case mix study at SNEC
Glaucoma	0.17	Dimensionless	Case mix study at SNEC
AMD	0.03	Dimensionless	Case mix study at SNEC
Myopia	0.02	Dimensionless	Case mix study at SNEC
Refractive error	0.02	Dimensionless	Case mix study at SNEC
ERM	0.01	Dimensionless	Case mix study at SNEC
RVO	0.01	Dimensionless	Case mix study at SNEC
Others	0.34	Dimensionless	Case mix study at SNEC
Population of completed care			
Cataracts	0.1	Dimensionless	Modelers assumption
Myopia	0.1	Dimensionless	Modelers assumption
Refractive error	0.1	Dimensionless	Modelers assumption
Average eye care visit per year	2.4	Visit/patient/year	Expert opinion
Ophthalmologist requirement module			
Medical school admission	Time series [2003–2013]	People	MOH
Medical school dropout rate	0	Dimensionless/year	
Fraction to other residency program	0.1	Dimensionless/year	Expert opinion
Attrition rate of house officers	0.1	Dimensionless/year	Expert opinion
Attrition rate of medical officers	Time series [2003–2013]	Dimensionless/year	SNEC data
Attrition rate of ophthalmologists	0.03	Dimensionless/year	Model calibration
Residency intake	Time series [2003–2014]	Persons/year	SNEC, NUH, NHG
Residency duration	5	Years	Expert opinion
Distribution of work hours			
Clinical	0.86	Dimensionless/year	Data from SNEC
Research	0.09	Dimensionless/year	Data from SNEC
Teaching	0.02	Dimensionless/year	Data from SNEC
Administration	0.03	Dimensionless/year	Data from SNEC
Visit-ophthalmologist-ratio: workload	Time series [2003–2014]	Visits/worker/year	MOH

### Model validation

For the model validation, two critical tests (behavior and structure validation test) were selected to demonstrate its fit and quality. The behavior test shows simulated behavior compared to available time series data of selected variables, demand, and number of ophthalmologists employed (refer to Additional file 1). The results indicate that the simulated model compares well with the time series data suggesting that on the face value, the model performs credibly for the visual fit test.

For the structure test, the model was presented to the stakeholders, to verify its structure and assumptions regarding causal relationships. Hence, the model is firmly grounded in current available evidence on the interactions between prevalence of eye conditions, utilization of eye care services, and the capacity of eye healthcare system.

### Scenarios

The following scenarios were developed to cover the range of potential future directions that were expressed by stakeholders:

Business-as-usual (BAU): The BAU scenario assumes no change to key variables that may be affected by policy change, i.e., uptake factor of eye services, current model of care, subsidies, and workload of eye care workforce remain unchanged from 2013 values up to 2040. Under this scenario, the uptake factor, which is calibrated, is 4.5% for individuals with no education, 7% for those with primary education, 7.6% for those with secondary education, and individuals with tertiary education is 15%. Also, it is assumed that under the new Primary Eyecare Clinic (PEC) initiative only 5% of all patients with DR, glaucoma, myopia, and refractive error are decanted from SOCs to PECs to be cared for by non-specialists. This hypothetical scenario is unlikely in the current context in which uptake factor is expected to change as the population becomes more aware of services and new models of care are introduced, including new technology, subsidies, and new care pathways. However, it is included to serve as a reference point for evaluating the alternative scenarios.

Current policy: This policy scenario is identical to the BAU scenario with the exception that the uptake factor among individuals with eye condition not receiving eye care is assumed to change. This is due to an expected increase in eye disease screening (which will significantly increase the number of people with diagnosed eye condition and consequently the use of services), awareness and availability of eye care clinics in the community, and new models of care including new technology which makes eye care more accessible and available. Thus, it is assumed that care seeking among individuals with untreated eye condition increases from 4.5% to 13% by 2040 for those with no education, while that for individuals with primary, secondary, and tertiary education are 20%, 21%, and 46%, respectively.

New model of care: The new model of care scenario is like the current policy scenario except that, under the new PEC initiative, 20% of all patients with DR, and glaucoma, as well as 90% of patients seeking care with myopia and refractive error, are decanted from SOCs to PECs to be cared for by non-specialists [30, 31]. The role of non-specialists (e.g., optometrists) running clinics is currently very limited (there is no bachelor education in optometry available in Singapore). Experiences in other countries such as the U.K. and Australia show, however, the potential of larger transitions.

Moderated workload: The moderated workload scenario is indistinguishable to the new model of care scenario with the exception of a 15% reduction in the clinical workload of ophthalmologists due to efforts to pursue non-clinical goals (such as research and education), improve work-life balance, and improve patient care in line with the focus on care delivery in an academic medical center setting.

#### Sensitivity analysis

Sensitivity analysis was performed using Markov chain Monte Carlo (MCMC) [32] on the base-case and other scenarios to observe how a change in the most important parameter affects output of interest. The uptake factor parameter was identified to be the most important parameter. Using MCMC, accepted points (after the optimal burn-in period) from the MCMC calibration were used as input to the sensitivity runs so as to explore the response of variables of interest in the model subject to the posterior probabilities from the calibration. The model was run 24 000 times. Next, the minimum and maximum values at 95% confidence level for each run were used to show the credible interval.

## Results

### Prevalence of eye diseases

Projections for the various eye conditions for resident Singaporeans 40 years and older up to the year 2040 can be seen in Table 2. Among the eye conditions, the prevalence of DR, other conditions, glaucoma, and ERM show the greatest increase from 2015 to 2040, increasing by 113%, 110%, 102%, and 97%, respectively. This is followed by cataract, myopia, AMD, RVO, and under-corrected refractive error, with increases of 82%, 70%, 55%, 47%, and 22%, respectively.

**Table 2:** **Prevalence of eye conditions for resident Singaporeans 40 years and older**

**Eye condition**	**Base year**	**Projected**						**% change from 2015 to 2040**
	**2010**	**2015**	**2020**	**2025**	**2030**	**2035**	**2040**	
Cataract	590 000	739 400	900 200	1 054 800	1 186 100	1 283 500	1 345 300	82
DR	92 000	116 500	144 100	172 700	200 300	225 800	248 600	113
Glaucoma	59 200	75 300	93 800	112 600	129 800	143 300	152 100	102
AMD	106 500	125 700	145 700	164 600	180 300	190 700	194 900	55
Myopia	957 000	1 148 100	1 317 900	1 483 700	1 654 800	1 818 600	1 954 900	70
Refractive error	323 600	359 400	385 500	403 500	416 900	428 300	438 600	22
ERM	217 100	276 100	340 900	405 000	461 600	508 200	544 800	97
RVO	12 600	14 300	15 900	17 300	18 700	20 000	21 000	47
Other conditions	73 500	90 500	109 900	131 400	153 900	174 600	190 400	110

In 2015, the three most prevalent conditions are myopia, cataract, and under-corrected refractive error, with approximately 1.15 million, 0.74 million, and 0.36 million cases, respectively. By 2040, myopia cases are projected to remain the most prevalent condition, with a total of 1.95 million cases. This is followed by cases of cataract and ERM, with 1.35 million cases of cataract and 0.54 million cases of ERM by 2040. In decreasing order of prevalence, the next conditions are under-corrected refractive error, DR, AMD, other conditions, glaucoma, and RVO, with numbers below 0.45 million.

### Demand

The projected demand for eye care services can be seen in Table 3. As expected, public sector demand for eye care services in Singapore are projected to increase the most under the current policy scenario from 2015 to 2040 (175%), followed by the new model of care and moderate workload scenarios from 2015 to 2040 (150%). By 2040, under the BAU scenario, patient visits are projected to increase 117% from 0.72 million (sensitivity analysis at 95% confidence range: 0.54 to 0.93 million) in 2015 to 1.56 million (0.97 to 1.87 million) in 2040. Under the current policy scenario, patient visits are expected to increase 175% from 0.73 million (0.6 to 0.9 million) in 2015 to 2.02 million (1.74 to 2.11 million) in 2040. With the new model of care and moderate workload scenarios, there is a 150% projected growth in patient visits from 0.73 million (0.67 to 0.91 million for new model of care and 0.67 to 0.92 million for moderated workload) in 2015 to 1.83 million (1.59 to 1.93 million for both) in 2040.

**Table 3:** **Projected eye care demand and required ophthalmologists up to 2040**

**Outcome**	**Base year**	**Projected**						**% change from 2015 to 2040**
	**2010**	**2015**	**2020**	**2025**	**2030**	**2035**	**2040**	
Eye care demand								
Business-as-usual	568 200 [445 000–765 700]	718 500 [537 500–927 500]	887 200 [613 600–1 113 500]	1 069 700 [709 400–1 330 200]	1 251 800 [804 200–1 538 700]	1 418 600 [891 400–1 724 100]	1 557 900 [971 100–1 866 800]	117%
Current policy	568 200 [471 200–734 700]	733 200 [603 800–905 600]	997 500 [828 800–1 166 500]	1 296 000 [1 061 500–1 445 700]	1 582 500 [1 302 400–1 715 600]	1 828 500 [1 536 400–1 939 000]	2 019 000 [1 737 300–2 112 800]	175%
New model of care	568 200 [526 200–746 000]	733 200 [666 100–914 300]	965 400 [853 400–1 148 300]	1 229 000 [1 058 500–1 395 000]	1 477 200 [1 259 600–1 621 000]	1 682 100 [1 449 400–1 801 600]	1 830 600 [1 592 900–1 929 800]	150%
Moderated workload	568 200 [526 200–749 500]	733 200 [666 400–917 900]	965 400 [850 000–1 150 900]	1 229 000 [1 054 300–1 399 500]	1 477 200 [1 256 100–1 624 200]	1 682 100 [1 446 900–1 803 900]	1 830 600 [1 592 900–1 930 700]	150%
Required ophthalmologists								
Business-as-usual	104 [88–152]	141 [125–216]	174 [143–260]	210 [166–310]	245 [188–359]	278 [208–402]	305 [227–436]	117%
Current policy	104 [95–134]	144 [131–178]	196 [173–229]	254 [220–283]	310 [268–336]	359 [312–380]	396 [351–414]	175%
New model of care	104 [96–136]	144 [131–179]	189 [167–225]	241 [208–274]	290 [247–318]	330 [284–353]	359 [312–378]	150%
Moderated workload	104 [96–137]	145 [131–181]	196 [173–234]	257 [221–293]	319 [271–351]	375 [323–402]	422 [367–445]	192%

### Workforce requirements

The number of ophthalmologists required can also be seen in Table 3. Under the BAU scenario, the projected number of ophthalmologists required by the year 2040 will increase 117% from 141 (sensitivity analysis at 95% confidence range: 125–216) in 2015 to 305 (227–436) by the year 2040. For the current policy scenario, 144 (131–178) ophthalmologists are projected to be needed in 2015 and 396 (351–414) in 2040; that is a 175% increase in the number of ophthalmologists required, which is 1.30 times as many as the BAU scenario in 2040.

Under the new model of care scenario, the public sector is projected to require 144 (131–179) ophthalmologists in 2015 and 359 (312–378) in 2040, representing a 150% increase and 1.18 times that of the BAU scenario in 2040. Under the moderated workload scenario, 145 (131–181) ophthalmologists are projected to be required in 2015 and 422 (367–445) in 2040, which is a 192% increase and 1.38 times as many as the BAU scenario in 2040.

## Discussion

The results from the analysis suggest that the number of Singaporean residents aged 40 years and older with eye diseases can be projected to more than double by 2040 with DR, glaucoma, and ERM estimated to increase the most. Accordingly, the demand for eye care services is conjectured to rise significantly under all the plausible scenarios. Hence, the demand for ophthalmologists is expected to rise.

The projected increase in the number of Singaporeans with eye diseases is due in part to the aging population and expected population increase. The total population of Singapore (both resident and non-resident) is assumed to reach 6.9 million by 2040 [33]. In line with this, the elderly population (individuals 60 years and older) is projected to more than double by 2040. The prevalence of eye conditions, in general, increases with age; hence, the increasing and aging Singaporean population is projected to have a higher prevalence of eye conditions.

An increase in the demand for eye care services is also anticipated due to factors including increasing access to healthcare in Singapore, a more highly educated future elderly population, and increasing screening for eye conditions. In addition, the Singapore government is enacting policies to make healthcare more widely accessible and affordable for the elderly, such as the Pioneer Generation Package giving them additional subsidies on healthcare and insurance [34]. In the area of eye care in particular, the Singapore government is supporting endeavors such as the launch of a Mobile Eye Clinic, which is intended to provide comprehensive eye care for senior citizens who are unable to access services due to physical or logistical restrictions [35]. The SNEC has also opened a number of satellite eye clinics to reduce both traveling and waiting times for patients [36]. These measures are likely to increase utilization of eye care services. Moreover, the future elderly population is expected to comprise a larger proportion with higher levels of education due to the government’s push for education. In 2002, 57.1% of males and 51.6% of females in Singapore had at least a secondary education. In 2012, the proportions of males and females with at least a secondary education rose to 70.8% and 64.9%, respectively [37]. The changing educational composition is likely to lead to higher visual acuity expectations, increasing further the utilization of eye care services.

Due to the increased demand for eye care services, the public sector eye care workforce requirements for ophthalmologists are projected to increase by 2040 for all the scenarios considered. To inform the provision of ophthalmology training without over or under supply of ophthalmologists, the number of required ophthalmologists to train to meet the projected demand was estimated, accounting for attrition.

Considering the uncertainties in the projections, under the BAU scenario, a residency intake of 8–22 residents per year is estimated as adequate to supply the required number of ophthalmologists in Singapore. Under the current policy scenario, a residency intake of 17–21 per year is projected to meet the required number of ophthalmologists. Under the new model of care scenario, an annual residency intake of 14–18 trainees is sufficient over the simulation time. When a moderate workload scenario is implemented, 18–23 residents per year will suffice.

### Limitations

One limitation of our projection of public sector visits and workforce requirements is that it is based on the assumption that the increase of public sector visits for all patients will be proportional to the increase in residents aged 40+ with eye conditions. Major changes in the proportion of visits by foreigners or by younger people would invalidate this assumption; however, foreigners and younger people represent a small proportion of all public sector visits. In addition, the projected demand depends on the projected demographic change in Singapore. Any significant changes observed in the population trend are likely to change the simulation results.

Further research on changes in age-specific prevalence rates, especially for myopia, would be highly desirable. Moreover, it would also be of interest to include the private sector provision of eye services in a future study; however, this would be a challenging task given the paucity of data on private care in Singapore. More importantly, further studies on the impact of educational attainment on use of eye care services are required.

## Conclusion

This paper provides a projection of a plausible future outlook for eye care in Singapore. The paper shows how and why the number of people with eye diseases and the demand for eye care services as well as workforce requirements will rise. The increase in demand is important to understand because it directly impacts the ability of the healthcare system to adequately and effectively provide eye care services for an aging and growing population.

In light of these results, human resource planners and policy makers should be aware of the potential value of analyzing and tracking over time the relationship between factors such as educational attainment, subsidies, changing visual acuity expectations, and new models of eye care provision on demand for eye care services. Policy makers will also benefit from a proactive approach that considers the effects of these factors on the uptake of services when planning human resource requirement for eye care services.

The systems modeling approach was useful in demonstrating the interdependence of the scenarios and system components and in providing policy makers with an overview of the levers available to them. Moreover, this model was designed to be sufficiently generic so that it could be applied to other countries in assessing future workforce requirements.

## Endnotes

^a^Data on number of ophthalmologists were taken from the Singapore Medical Council. Data for years 2007, 2008, and 2011 were unavailable.

^b^Data on patient visits per ophthalmologist were obtained from SNEC and used as a proxy for the whole of Singapore. Data were available for all years as shown.

^c^For the case mix administrative data from SNEC, refractive error refers to refractive error other than myopia.

^d^Other conditions include the SEED study categories of amblyopia, corneal conditions, PCO, pterygium, retinal scar, retinal dystrophy, optic disc, no obvious, aphakia, phthisis, trauma, squint, and others, an open category that includes all other eye diseases not classified into the previous 21 categories.

## Additional file

Additional file 1:
**Model validation for selected variables. a** Number of ophthalmologists, **b** demand for eye care.

## Abbreviations

AMD: age-related macular degeneration
BAU: business-as-usual
DR: diabetic retinopathy
ERM: epiretinal membrane
FTE: full-time equivalent
MCMC: Markov chain Monte Carlo
MOH: Ministry of Health
NHG: National Healthcare Group
NUH: National University Hospital
PEC: Primary Eyecare Clinic
RVO: retinal vein occlusion
SEED: Singapore Epidemiology of Eye Diseases
SNEC: Singapore National Eye Centre
SOC: specialist outpatient clinic
