# Supplementary figures and images for: Future requirements for and supply of ophthalmologists for an aging population in Singapore
Source: Hum Resour Health. 2015 Nov 17;13:86. doi: 10.1186/s12960-015-0085-4 (PMC4650855; doi:10.1186/s12960-015-0085-4)

## Ophthalmologists

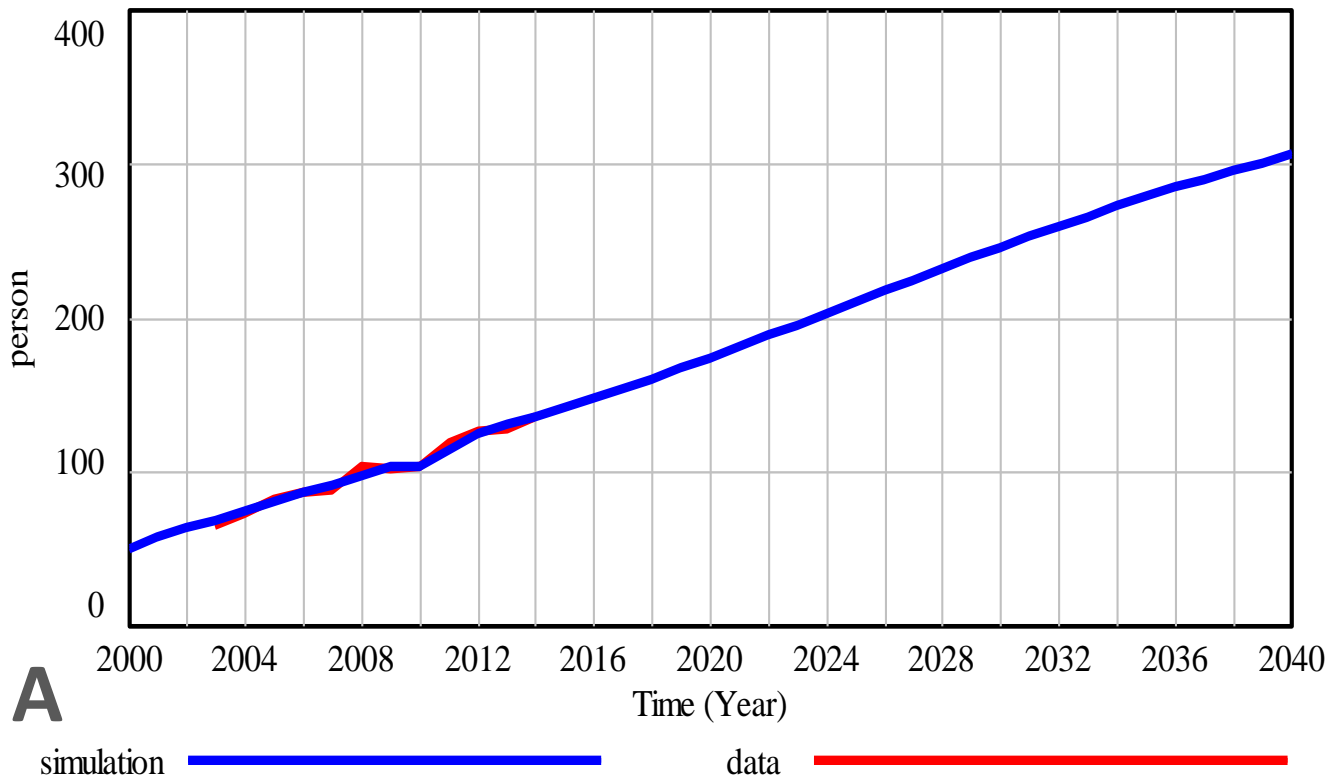

## Demand for Eye Care

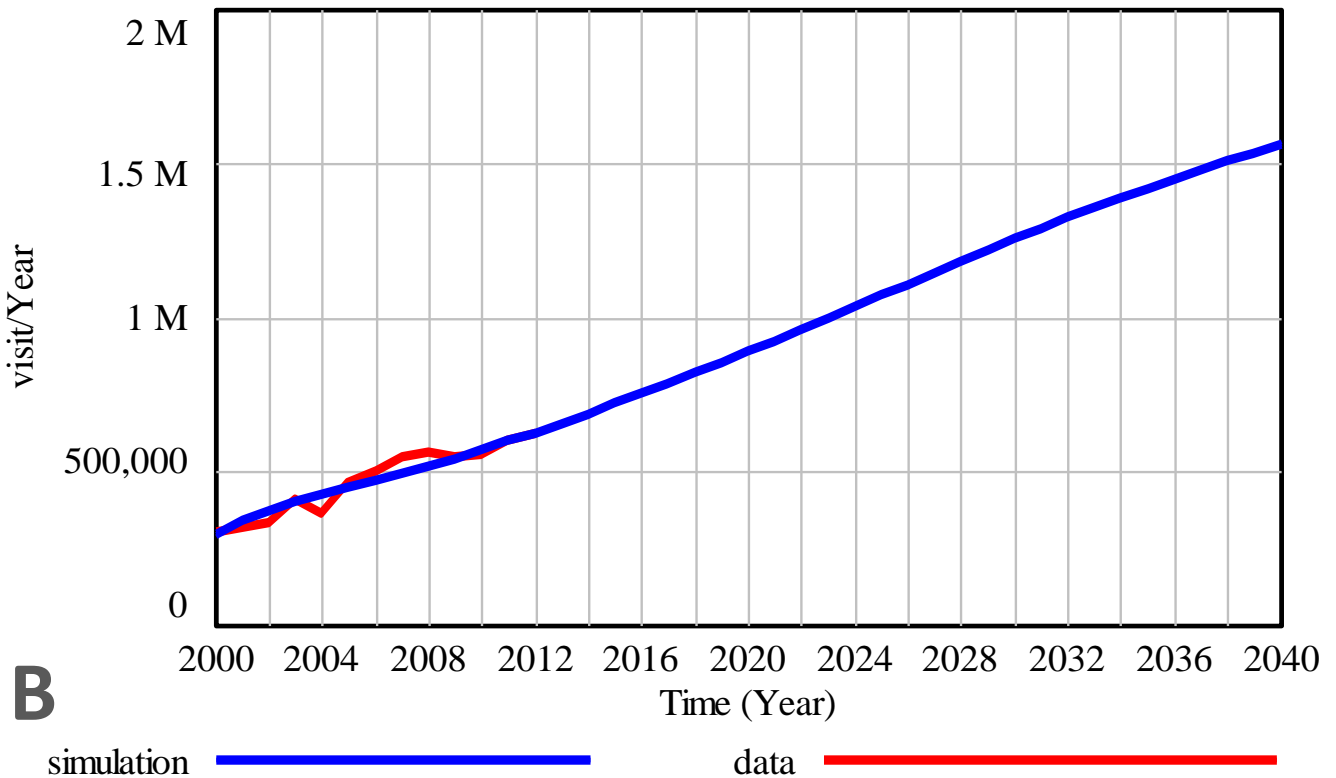

Supplement: Additional file 1: — Model validation for selected variables. a Number of ophthalmologists, b demand for eye care. [file 12960_2015_85_MOESM1_ESM.pdf]
